# Army Veterinary Department

**Published:** 1901-12

**Authors:** 


### ARMY VETERINARY DEPARTMENT.

From *The London Gazette:*

WAR OFFICE, PALL MALL, July 24.

The King has been graciously pleased to give orders for the following promotions . . . in recognition of the services of the undermentioned officers during the recent operations in China. Except where otherwise stated these rewards will bear date of November 29, 1900:

. . . . . .

### BREVET.

To be Veterinary-Major: Veterinary Captain E. H. Hazleton.

WAR OFFICE, July 30.

I. Y.—Surrey.—F. R. Brandt, gent., to be Veterinary-Lieutenant.

[In the United States Army the veterinarian has no rank, no future, and no social status.—ED.]

